# Physiological and Microbial Community Dynamics in Does During Mid-Gestation to Lactation and Their Impact on the Growth, Immune Function, and Microbiome Transmission of Offspring Kids

**DOI:** 10.3390/ani15070954

**Published:** 2025-03-26

**Authors:** Haidong Du, Kenan Li, Wenliang Guo, Meila Na, Jing Zhang, Jing Zhang, Renhua Na

**Affiliations:** 1 College of Animal Science, Inner Mongolia Agricultural University, Hohhot 010018, China; duhaidong1110@163.com (H.D.); 18686197338@163.com (W.G.); 15548710467@163.com (M.N.); zhangjing230518@163.com (J.Z.); 2 Grassland Research Institute of Chinese Academy of Agricultural Sciences, Hohhot 010010, China; likenan0826@yeah.net; 3 Intellectual Property Protection Center of Inner Mongolia Autonomous Region, Hohhot 010050, China; jingyuer96@163.com

**Keywords:** microbiome, gestation, lactation, immune response, metabolism, hormones, vertical transmission

## Abstract

This study investigated changes in physiological processes and rumen microbial communities in does from mid-gestation to lactation and identified potential associations between these physiological changes and the rumen microbiome. Additionally, we studied the transmission mechanisms of microorganisms between the dam and offspring. Our study demonstrates significant changes in maternal physiological metabolism, immune status, and rumen microbiota from mid-pregnancy through lactation. We identified potential associations between these physiological changes and the rumen microbiome. Moreover, the findings highlight that alterations in maternal physiological metabolism and immune status significantly influence the growth and immune development of offspring kids. Additionally, we observed that the maternal microbiota serves as a key source of gastrointestinal microbial communities in young animals, with early colonization of maternally derived microbes in the offspring’s gastrointestinal tract playing a role in shaping their immune system development. The results for primary outcomes are as follows: The serum levels of estrogen and progesterone in pregnant does were greater than those observed during lactation, while the concentration of growth hormone, triiodothyronine, and glucose exhibited an upward trend during lactation. During late gestation, the serum IL-10 concentration in does decreased, while the TNF-α concentration increased. Additionally, on day 140 of gestation, does showed a significant decrease in IgG, total protein, and globulin levels. From mid-gestation to lactation, the abundance of dominant phyla and genera, including Firmicutes, Bacteroidetes, *Patescibacteria*, *Bacteroidales_RF16_group*, *Clostridia_UCG-014*, *RF39*, and *Eubacterium_ventriosum_group*, in the rumen of does underwent significant changes. LEfSe analysis identified a series of marker microorganisms in the rumen of does at different physiological stages. A correlation was observed between these dominant bacteria and the serum physiological indicators of the does. Notably, rumen volatile fatty acids also exhibited a correlation with serum physiological indicators. In addition, serum physiological indicators of does were significantly correlated with the growth and immune indicators of their kids. Microbiological origin analysis revealed that the gastrointestinal microbiome of kids primarily originated from the rumen, birth canal, and milk of does. Further analysis identified a correlation between the kids’ serum immunometric indicators and certain gastrointestinal microorganisms. In particular, the jejunum microbiota of 28-day-old lactating kids, including *Alysiella*, *Neisseria*, and *Muribaculaceae*, showed a significant positive correlation with serum IL-6 and IL-10 levels. Meanwhile, these genera were dominant in the saliva and milk of does, suggesting a direct microbial transfer from dam to offspring. These microbial communities may play a significant role in modulating the metabolism and immune responses of the offspring, thereby influencing their immune system development.

## Simple Summary

This study explored how the health and rumen bacteria of does change from mid-pregnancy to the post-parturition period and how these changes affect their kids’ growth, immune system, and gastrointestinal bacteria. This study found that hormone levels in the doe, such as estrogen and progesterone, were higher during pregnancy than after birth, while other hormones and glucose increased after birth. During late pregnancy, the levels of certain immune factors in the serum of doe changed, with a decrease in anti-inflammatory factors and an increase in pro-inflammatory factors. The study also showed that the types of bacteria in the doe’s rumen changed significantly from mid-pregnancy to after birth, and these bacteria were linked to the doe’s overall health. Importantly, the health of the doe was connected to the growth and immune system of their kids. We discovered that the gastrointestinal bacteria of kids mainly originate from their mother’s rumen, birth canal, and milk. This finding help us understand how the health of does influences their kids and could lead to better ways to care for goats, improving their health and productivity, which is valuable for farming and food production.

## 1. Introduction

The reproductive performance and health of the dams directly influence the overall productivity and economic benefits of livestock farms. Gestation and lactation are two critical physiological transition phases in the life cycle of a dam. During this period, the physiological metabolism and immune status of the dam undergo significant changes. These adjustments in physiological and immune functions are not only crucial for maintaining maternal health but also have lasting effects on the growth and health of the offspring [1,2]. In pregnant goats, as gestation progresses, the concentration of GH in the maternal circulation consistently increases [3,4]. This rise in GH levels subsequently elevates glucose concentrations in the maternal circulation and enhances the placenta’s ability to transfer nutrients to the fetus, thereby influencing fetal growth and development [5]. Fetal thyroid hormone primarily depends on maternal supply via the placenta and plays a critical role in the development and maturation of fetal tissues and organs. Beginning in mid-gestation, maternal thyroid hormone levels gradually rise, peaking during late gestation, thereby ensuring an adequate supply of thyroid hormone to the fetus [3,6]. To meet the glucose demands of both the mother and the fetus during late gestation, the mother secretes large amounts of hormones that induce insulin resistance, such as glucagon, growth hormone, and cortisol. This results in a state of physiological insulin resistance, reducing the sensitivity of insulin target organs and ensuring an adequate glucose supply [7,8]. Concurrently, insulin resistance promotes fat breakdown, shifting the mother’s metabolism to a catabolic state where fat is preferentially used as an energy source, thus preserving glucose for the fetus [9,10]. Most animals transition to a state of higher energy demand after giving birth. During lactation, the mother mobilizes her fat reserves and enhances hepatic metabolic function to meet the energy requirements for milk production. However, excessive fat mobilization within liver cells during this period can lead to maternal metabolic disorders and inflammation [11]. Additionally, the maternal body composition is transmitted to the offspring through milk, influencing the microbial colonization of the offspring’s gut [12]. For instance, milk oligosaccharides promote the colonization of Bifidobacterium and Bacteroides in the gut of the offspring [13]. Zou et al. found that the pathogenic bacterium Mannheimia rapidly colonizes the rumen of lambs after birth but disappears once the lambs receive milk, likely due to the presence of antibodies and immune factors in colostrum that can counteract Mannheimia [14].

During this physiological process, the gastrointestinal microbiota as the “second genome” of the host, through its dynamic adjustment of structure and function serves an integral role in maternal health and offspring development [15]. There is evidence to suggest that the maternal microbiota is not in a static state but rather a complex ecosystem that can dynamically respond to various physiological changes during gestation and lactation [16,17]. During the later stages of gestation, elevated levels of progesterone in the mother’s body correspondingly promote an increase in intestinal *Bifidobacterium* abundance. *Bifidobacterium* may benefit maternal health by regulating weight gain, improving insulin sensitivity, and enhancing immune function [18]. Koren et al. found that a high abundance of Proteobacteria and Actinobacteria in the maternal gut during late pregnancy resulted in reduced insulin sensitivity [17]. Furthermore, changes in the maternal microbiome during pregnancy can influence metabolic pathways, thereby altering the nutritional availability for the developing fetus [19]. The maternal immune system is in an immune adaptation status during gestation to support fetal development while simultaneously preventing immune rejection. The immune adaptation of the dam is closely associated with the microbiota, which likely play key roles in inducing a tolerant immune response, thereby protecting the fetus from potential attacks by the maternal immune system [20,21,22]. Furthermore, it has been shown that *Faecalibacterium* and *Eubacterium* (butyrate-producing bacteria) counts increase in the maternal gut during early gestation [17]. Butyrate possesses anti-inflammatory and immunomodulatory properties and plays a crucial role in the establishment of pregnancy by modulating T cells to mitigate inflammation within the reproductive system [23]. Changes in the maternal microbiome during lactation are equally crucial for the health of both the dam and her offspring. During lactation, the maternal gastrointestinal microbiome plays an important role in sustaining metabolic homeostasis, supporting the development of the neonatal immune system, and ensuring successful gut microbiome colonization [24]. Previous research has demonstrated that during lactation, the abundance of *Ruminococcus* and *Fibrobacter* increases in the mother, and these bacteria ferment carbohydrates to produce volatile fatty acids (VFAs), which contribute to meeting the energy demands of lactation [25,26]. At the same time, it has been reported that when the young animals are reared together with their dam, the nutrients, microorganisms, and immune regulatory factors of the dam can be efficiently transferred to the young animals, thus facilitating the development of the microbial community and immune function of the young animals [27,28]. When maternal microorganisms colonize the offspring’s intestinal tract, they provide antigenic stimulation to the host, triggering the differentiation of regulatory T cells. This process induces gene expression in the host, resulting in the upregulation of genes associated with intestinal immunity and development. Consequently, it drives the development of the host’s intestinal tract and the establishment of its immune system [29,30,31,32,33].

Immune maturation and microbial colonization in young animals significantly impact their long-term health. In summary, maternal physiological activities, immune status, and the microbial composition during gestation and lactation have significant and far-reaching impact on the health of both the dam and the offspring. However, systematic study examining the relationship between the physiological metabolism, immune status, rumen microbiome of does, and their effects on maternal and neonatal health, kid microbiome colonization, and immune development is still lacking. The period from mid-gestation to lactation is a time of rapid increase in the nutritional needs of the doe. During this phase, there is not only a profound and rapid alteration in maternal physiological metabolism to support fetal development and meet the demands of lactation, but it is also a critical stage for rapid fetal growth and postnatal development of the offspring. Therefore, this study selected cashmere goats as the research subject and analyzed the changes in hormones, immunity, metabolism, and rumen microbiota in does from mid-gestation to lactation as well as the mechanisms underlying the interactions of these changes. In the meantime, we also analyzed the maternal factors that influence microbial colonization and immune development in newborn and suckling kids, with the aim of providing a reference for the management of does and kids during these critical periods.

## 2. Materials and Methods

### 2.1. Does Feeding Experiment and Sampling

Before the trial began, we performed estrus synchronization and artificial insemination on the does. At 60 days of pregnancy, a total of 18 multiparous Inner Mongolia cashmere goats with similar body weights (age = 4; initial BW = 46.09 ± 2.83 kg; parity= 3) were selected as experimental animals for this study. Animals were assigned to three completely covered dirt pens (six does/pen) with automatic watering systems. This experimental design included three pen replicates (one pen per treatment group) and 18 biological replicates (six does per pen). During the experimental period, the nutritional requirements for cashmere goats met or exceeded the nutrient requirements of cashmere goats (NY/T 4048-2021) [34], as shown in Table S1. The trial lasted from day 60 of gestation to day 28 of lactation. The does were fed twice daily, at 8:00 a.m. and 4:00 p.m. and had ad libitum access to water. After delivery, the kids and does were maintained in the same pens, and the does breastfed kids, ensuring none of the kids had an opportunity to obtain the does’ feed. The treatment group consisted of 18 does, including 8 with twin pregnancies and 10 with single pregnancies. To ensure each doe nursed only one kid, one of the twins was removed shortly after birth in the cases of twin births. As a result, the group comprised 11 male kids and 7 female kids. The birth weight and the weight of the kids at 28 days of age were recorded.

Based on the growth patterns of fetuses, the physiological and metabolic changes in does, and the characteristics of microbial colonization in newborn kids, we selected specific sampling time points: 75, 105, and 140 days of gestation as well as 0 and 28 days postpartum. On days 75 (G75), 105 (G105), and 140 (G140) of gestation as well as on days 0 (L0) and 28 (L28) of lactation, two does per pen (totaling six does across three pens) with body weights close to the group average were selected for sampling. From each selected doe, 10 mL of blood was collected from the jugular vein. From each pen, two does with body weights close to the group average were selected (totaling six does), and 10 mL of blood was collected from the jugular vein of each doe. The blood samples were then centrifuged at 3000× *g* at 4 °C for 15 min to obtain serum. The serum was subsequently stored at −80 °C for biochemical factor analysis. Subsequently, two does were selected from each of the three pens, and approximately 50 mL of rumen fluid was collected from each doe through a stainless-steel stomach tube using a rumen vacuum sampler. After delivery, sterile swabs were used to collect samples from the breast skin, birth canal, and rectum and the surrounding environment; sterile sponges were used to collect saliva from the does. Additionally, at 28 days postpartum, doe saliva, breast skin samples, and rectal fecal samples were also collected. Samples were collected from the ground and railings of the goat pens using sterile swabs. The purpose was to exclude contamination from microorganisms in the goat pen environment on the microbial samples taken from various parts of the doe’s body. After cleaning the breast with an alcohol swab and sterile gauze, milk was collected manually. Microbial samples from the enclosure environment were mainly used as negative controls. On postnatal day 0 (within 10 min of birth) and day 28, twelve kids were euthanized after the collection of blood samples. The abdominal cavity was then opened, and sterile pipette and sampling spoons were used to collect rumen mucus and meconium from the 0-day-old kids as well as rumen and jejunal contents from the 28-day-old kids. These samples were transferred into sterile cryovials. The collected samples from the does and kids were stored at −80 °C for microbiological analysis.

### 2.2. Serum Parameters

The serum levels of total protein (TP), albumin (ALB), globulin (GLB), triglyceride (TG), high-density lipoprotein cholesterol (HDL-C), and glucose (Glu) were measured by the commercial kits (Beijing Lepu Diagnostics Co., Ltd., Beijing, China). An ELISA kit (Beijing Huaying Biotechnology Research Institute, Beijing, China) was used to measure the levels of serum progesterone (P), estrogen (E2), growth hormone (GH), insulin-like growth factor 1 (IGF-1), triiodothyronine (T3), and thyroxine (T4). The levels of interleukin-6 (IL-6), interleukin-10 (IL-10), tumor necrosis factor-α (TNF-α), IgA, IgG, IgM, lipopolysaccharide (LPS), and diamine oxidase (DAO) in doe serum as well as IL-6, IL-10, TNF-α, IgA, IgG, and IgM in kid serum were measured using ELISA kits (Shanghai Enzyme-linked Biotechnology Co., Ltd., Shanghai, China).

### 2.3. DNA Extraction, 16S rRNA Sequencing, and Data Analysis

Microbial genomic DNA was extracted from does (saliva, rumen, breast milk, skin, birth canal, and fecal samples), kids (rumen, meconium, and jejunal contents), and enclosure environment samples using the Magnetic Soil and Stool DNA Kit (TianGen, Beijing, China, Catalog #: DP712).

DNA purity and concentrations were assessed by agarose gel electrophoresis and UV spectrophotometry. The V4 region of the bacterial 16S rRNA gene was amplified by PCR with universal primers (515F and 806R), as previously described [35]. Library construction and sequencing was performed by Novogene with Illumina NovaSeq 6000 platform (Novogene Bioinformatics Technology Co., Ltd., Beijing, China).

The paired-end Illumina reads were subjected to adapter trimming and quality filtering using fastp software (v0.23.1). Reads with lengths shorter than 50 bp, quality values below 20, or containing N bases were removed. Subsequently, chimeric sequences were identified and eliminated using Vsearch (Version 2.15.0). Amplicon sequence variants (ASVs) were generated through denoising with DADA2, followed by the removal of ASVs with an abundance of less than 3. ASVs were annotated using the Silva (v138) database [36]. In this study, a total of 109 microbial samples were collected and sequenced on the Illumina NovaSeq 6000 platform by Novogene. The sequencing process yielded 13,495,984 raw reads. After the removal of invalid reads, 12,088,377 high-quality reads were retained, with an average of 110,902 reads per sample.

### 2.4. Volatile Fatty Acids Determination

Then, 1 mL of rumen fluid was placed into bottles containing 0.2 mL of 25% metaphosphoric acid and centrifuged at 10,000× *g* for 15 min at 4 °C to obtain a supernatant. The supernatant was filtered with 0.22 μm aqueous filter membrane; after this, 1 µL filtrate was injected through a micro-injector into the gas chromatograph (Clarus680, PerkinElmer, Waltham, MA, USA). Temperature programming of the column: the initial temperature was 110 °C and heated at the speed of 10 °C/min to 150 °C, where it remained for 5 min.

### 2.5. Statistical Analysis

For doe and kid serum data, Student’s *t*-test was used to compare two groups of the data, while one-way ANOVA was used to compare multiple groups of data (SAS software, v.9.2, SAS Institute, Cary, NC, USA). Data are shown as mean ± standard deviation (STDEV). *p*-value ≤ 0.05 was considered statistically significant for all comparisons.

Statistics were calculated through QIIME2 (alpha diversity: Shannon and Chao 1 index, Kruskal–Wallis tests, and Spearman rank correlations). Statistical significance of differences between the groups were determined by multiple response permutation procedure (MRPP) and analysis of group similarities (ANOSIM). Principal coordinate analysis (PCOA) analysis based on Bray–Curtis distance was conducted with R software (version 3.6.1). The microbial data were statistically analyzed using a Kruskal–Wallis H test. *p*-values were corrected for multiple testing using Benjamini–Hochberg. Linear discriminant analysis effect size (LEfSe) identified microbial biomarkers among groups (LDA > 3, *p* < 0.05). Source Tracker software (https://cloud.majorbio.com/page/tools/, accessed on 11 December 2024) was utilized to predict potential sources of the GI microbiota in lambs. Permutational multivariate analysis of variance (PERMANOVA) was used to assess the relationship between the microbiota of the does and the gastrointestinal microbiota of the kids. Prior to PERMANOVA analyses, the data were tested to ensure equal dispersion using a resemblance-based permutation test (PERMDISP).

## 3. Results

### 3.1. Changes in the Serum Physiological Indices of Does During Mid-Gestation and Lactation

**Figure 1:**
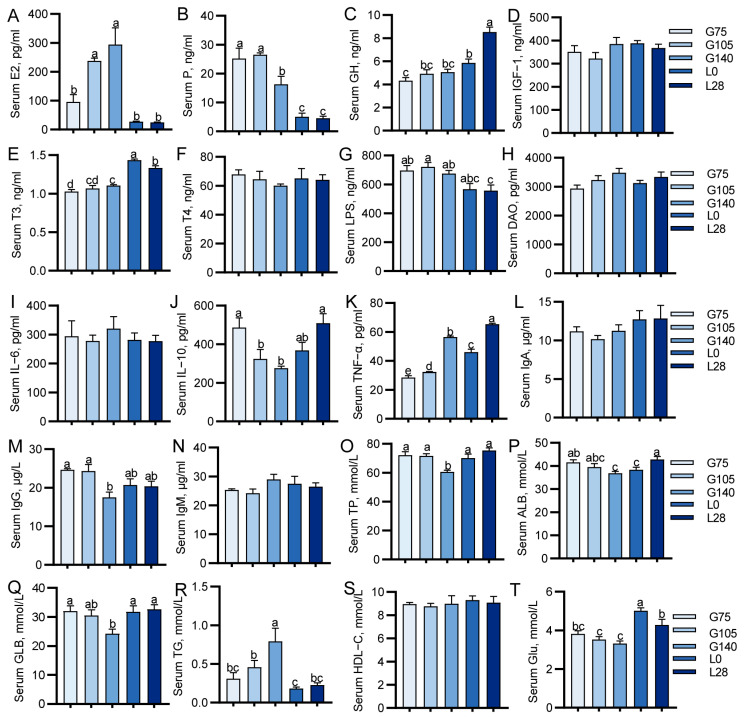
Changes in the serum physiological indices of does during mid-gestation and lactation. (**A**–**F**) Hormonal indicators, (**G**–**N**) immune indices, and (**O**–**T**) metabolic indicator. G75, G105, and G140: days 75, 105, and 140 of gestation; L0 and L28: days 0 and 28 of lactation. TP, total protein; ALB, albumin; GLB, globulin; TG, triglyceride; GLU, glucose. Different letters represent significant differences (*p* < 0.05). *n* = 6/stage.

To identify physiological changes in does during gestation and lactation, we conducted a systematic evaluation of hormonal fluctuations, inflammatory responses, and metabolic processes throughout these stages. The highest levels were found in serum E2 levels in G105 and G140 does (*p* < 0.01; Figure 1A). Serum P levels were highest in G75 and G105 does and then gradually decreased (*p* < 0.01; Figure 1B). A stepwise increase in GH levels was observed between G75 and L28 (*p* < 0.01; Figure 1C). Serum T3 exhibited a gradual increase from G75 to G140, and serum T3 levels were significantly higher during lactation (L0 and L28) compared to the gestation period (*p* < 0.01; Figure 1E). Serum LPS levels in pregnant does (G75, G105, and G140) were found to be significantly higher than those in L28 does (*p* < 0.05; Figure 1G). Serum IL-10 levels in G105 and G140 does were significantly lower than in G75 and L28 does (*p* < 0.01; Figure 1J). Serum TNF-α levels increased significantly from G75 to G140, reaching the highest levels in both G140 and L28 does (*p* < 0.01; Figure 1K). Serum levels of IgG, TP, and GLB were significantly lower in G140 does than in the other four periods (*p* < 0.05; Figure 1O,M,Q). Serum ALB levels in G140 and L0 does were significantly lower than those in G75 and L28 does (*p* < 0.05; Figure 1P). G140 had the highest serum TG levels (*p* < 0.01; Figure 1R). Serum glucose levels during lactation exhibited a significant increase compared to those during gestation (*p* < 0.01; Figure 1T). No notable differences were identified in the serum concentrations of IGF-1, T4, DAO, IL-6, IgA, IgM, and HDL-C (*p* > 0.05; Figure 1D,F,H,I,L,N,S).

### 3.2. Microbiological Analysis of the Rumen Microbiota in Does During Mid-Gestation and Lactation

**Figure 2:**
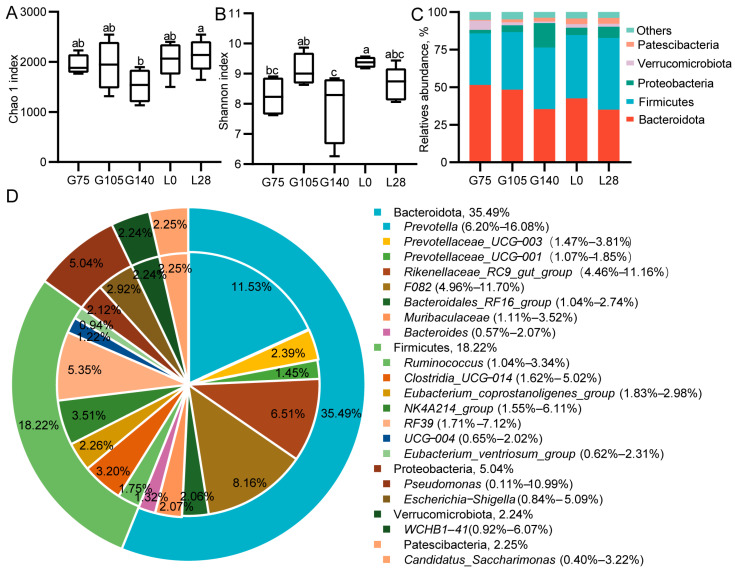
Microbiological analysis of the rumen microbiota in does during mid-gestation and lactation. (**A**–**B**); α diversity–Chao1 and Shannon indexes. (**C**), The relative abundances of the top five phylum during gestation and lactation. (**D**) The relative abundances of the top 19 genera during gestation and lactation. Different letters represent significant differences (*p* < 0.05). G75, G105, and G140: days 75, 105, and 140 of gestation; L0 and L28: days 0 and 28 of lactation. *n* = 6/stage.

To examine changes in the rumen microbiome structure in does during mid-gestation and lactation, 16S rRNA sequencing was conducted on the rumen microbiome. We performed sparse analysis on the sequencing samples and observed a curved plateau (Figure S1), indicating that the sequencing depth was adequate. Compared to the other four time points, the Chao1 index and the Shannon index had their lowest values at G140 (*p* < 0.05; Figure 2A,B). The ANOSIM and MRPP results demonstrated that there were notable differences in the structure of the rumen microbiota between gestation and lactating does (*p* < 0.05; Table S2). At the phylum level, the rumen microbiota of does during gestation and lactation was primarily dominated by Firmicutes, Bacteroidetes, Proteobacteria, Verrucomicrobia, and Patescibacteria (Figure 2C). At the genus level, *Prevotella*, *F082*, *Rikenellaceae_RC9_gut_group* (Bacteroidetes), and *RF39* (Firmicutes) were the four most dominant genera in the rumen of pregnant and lactating does (Figure 2D).

### 3.3. Changes in the Dominant Bacterial Genera in the Rumen of Does During Mid-Gestation and Lactation

**Figure 3:**
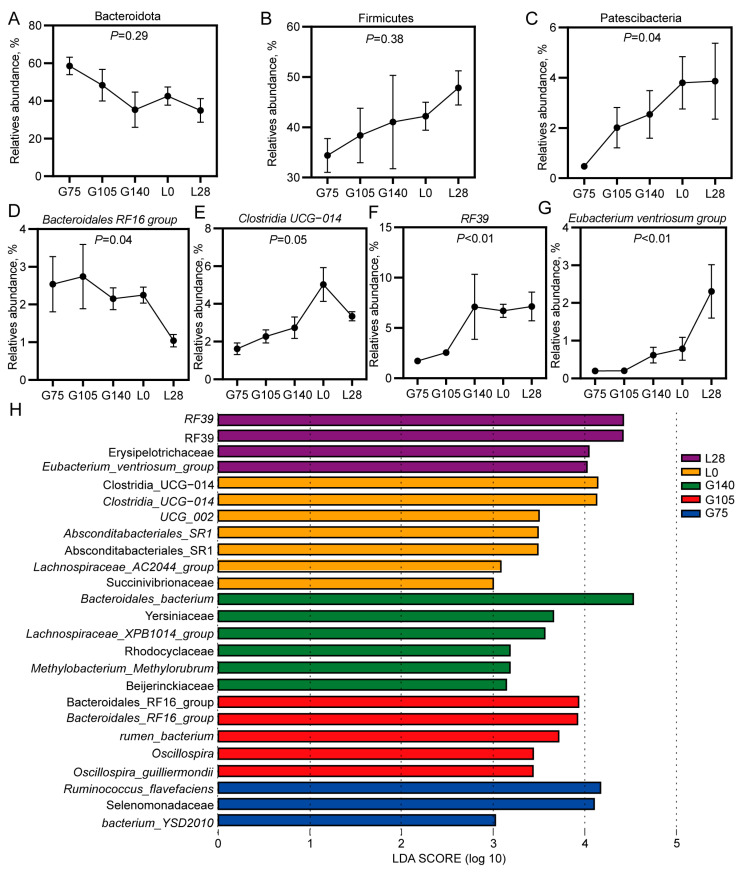
Changes in the dominant bacterial genera in the rumen of doe during mid-gestation and lactation. (**A**–**C**) The shifts in dominant phyla. (**D**–**G**) The shifts in dominant genus. (**H**) LEfSe analyses were used to identify the rumen microbial community structure (LDA > 3). G75, G105, and G140: days 75, 105, and 140 of gestation; L0 and L28: days 0 and 28 of lactation. *n* = 6/stage.

To investigate the changes in dominant bacteria during gestation and in lactating does, we conducted a differential analysis of the dominant bacteria. At the phylum level, from G75 to L28, the abundance of the Bacteroidetes showed a decline, while the abundance of the Firmicutes exhibited an increase. However, these differences were not statistically significant (*p* > 0.1; Figure 3A,B). The abundance of *Patescibacteria* showed a significant linear increase from G75 to L28 (*p* < 0.05; Figure 3C). The abundance of the *Bacteroidales_RF16_group* showed a significant linear decrease from G75 to L28, with the highest abundance observed at G105 (*p* < 0.05; Figure 3D). The abundance of *Clostridia_UCG-014* increased linearly from G75 to L0 (*p* = 0.05; Figure 3E). The abundance of *RF39* increased linearly from G75 to G140 (*p* < 0.01; Figure 3F). The abundance of *Eubacterium_ventriosum_group* increased linearly from G75 to L28 (*p* < 0.01; Figure 3G).

We employed LEfSe analysis to identify key microbial populations responsible for the differences in rumen microbiota structure between pregnant and lactating does (LDA > 3; Figure 3H). *Ruminococcus flavefaciens*, *Selenomonadaceae*, and the *bacterium YSD2010* were found to be enriched in the rumen of G75 does. The rumen of G105 does was mainly enriched with *Bacteroidales_RF16_group*, *rumen_bacterium*, *Oscillospira*, and *Oscillospira guilliermondii*. The rumen of G140 does was primarily enriched with *Bacteroidales_bacterium*, *Yersiniaceae*, *Lachnospiraceae_XPB1014_group*, *Rhodocyclaceae*, *Methylobacterium_Methylorubrum*, and *Beijerinckiaceae*. The rumen of L0 does was predominantly enriched with *Clostridia UCG_014*, *UCG_002*, *Absconditabacteriales_SR1*, *Lachnospiraceae_AC2044_group*, and *Succinivibriaceae*. Meanwhile, L28 was mainly enriched with *RF39*, *Erysipelotrichaceae*, and the *Eubacterium_ventriosum_group*.

### 3.4. Correlation Analysis Between Phenotypic Indicators and Microorganisms in Does

**Figure 4:**
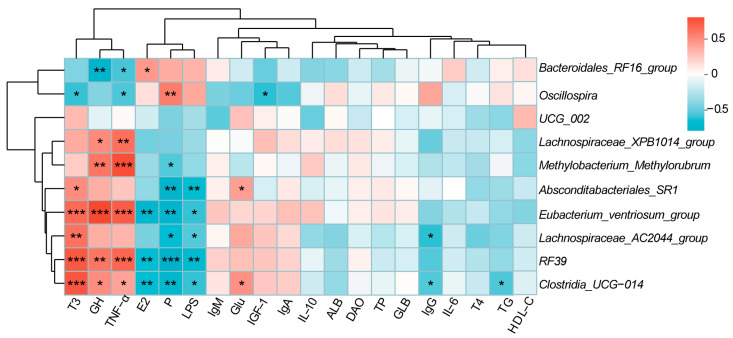
Correlation analysis between phenotypic indicators and microorganisms in does. * *p* < 0.05, ** *p* < 0.01, and *** *p* < 0.001. *n* = 6/stage.

We further performed Spearman’s correlation analysis between the microbiome and serum hormones, immune factors, and metabolic markers (Figure 4). The relative abundance of *RF39* and *Eubacterium_ventriosum_group* was significantly positively correlated with serum levels of T3 (r = 0.70, r = 0.64; *p* < 0.01), GH (r = 0.59, r = 0.77; *p* < 0.01), and TNF-α (r = 0.65, r = 0.69; *p* < 0.01) and significantly negatively correlated with levels of E2 (r = −0.52, r = −0.59; *p* < 0.01), P (r = −0.64, r = −0.60; *p* < 0.01), and LPS (r = −0.55, r = −0.48; *p* < 0.05). *Clostridia_UCG-014* was significantly positively correlated with serum T3 (r = 0.71, *p* < 0.001), GH (r = 0.50, *p* < 0.05), TNF-α (r = 0.41, *p* < 0.05), and Glu (r = 0.47, *p* < 0.05) and significantly negatively correlated with E2 (r = −0.53, *p* < 0.01), P (r = −0.59, *p* < 0.01), LPS (r = −0.48, *p* < 0.05), IgG (r = −0.41, *p* < 0.05), and TG (r = −0.42, *p* < 0.05). *Lachnospiraceae_AC2044_group* exhibited a significant positive correlation with serum T3 (r = −0.61, *p* < 0.01) and a significant negative correlation with P (r = −0.51, *p* < 0.01), LPS (r = −0.41, *p* < 0.05), and IgG (r = −0.48, *p* < 0.05). *Absconditabacteriales_SR1* had a positive association with T3 (r = 0.50, *p* < 0.05) and Glu (r = 0.43, *p* < 0.05) and a negative association with P (r = −0.60, *p* < 0.01) and LPS (r = −0.52, *p* < 0.01). *Lachnospiraceae_XPB1014_group* and *Methylobacterium_Methylorubrum* were significantly positively correlated with serum GH (r = 0.51, r = 0.59; *p* < 0.05) and TNF-α (r = 0.62, r = 0.73; *p* < 0.01) (*p* < 0.05). *Methylobacterium_Methylorubrum* also showed a significant negative correlation with serum P (r = −0.41, *p*< 0.05). *Oscillospira* was significantly positively correlated with P (r = 0.56, *p* < 0.01) and significantly negatively correlated with T3 (r = −0.48, *p* < 0.05), TNF-α (r = −0.42, *p* < 0.05), and IGF-1 (r = −0.48, *p* < 0.05). Finally, *Bacteroidales_RF16_group* was significantly negatively correlated with GH (r = −0.60, *p* < 0.01) and TNF-α (r = −0.44, *p* < 0.05) levels and significantly positively correlated with E2 levels (r = 0.45, *p* < 0.05).

### 3.5. Changes in the Level of Volatile Fatty Acids in the Rumen of Doe During Mid-Gestation and Lactation

According to Table 1, the acetate concentrations in the rumen exhibited an overall upward trend during mid-gestation (G75), late gestation (G105), and lactation (L0 and L28). Specifically, the acetate concentrations in the rumen of L28 does were found to be significantly higher than that in G75 and G140 does (*p* < 0.05). The acetate concentrations in the rumen of G105 does was significantly higher than those in G75 does (*p* < 0.05). At the five time points during gestation and lactation, there was no significant change in the rumen VFA content of does, except for acetate. We also analyzed the correlation between serum indicators and rumen VFAs (Table S3). The results showed that acetate was positively correlated with T3 (r = 0.44) and GH (r = 0.47; *p* < 0.05). Propionate was significantly negatively correlated with E2 (r = −0.59), LPS (r = −0.49) and TG (r = −0.63) and significantly positively correlated with GH (r = 0.44) and Glu (r = 0.54, *p*< 0.05). A significant positive correlation was observed between butyrate and TP (r = 0.52, *p* < 0.05). Isobutyrate was significantly positively correlated with IGF-1 (r = 0.52, *p* < 0.05). Furthermore, TVFA was significantly negatively correlated with E2 (r = −0.57) and TG (r = −0.65) and significantly positively correlated with Glu (r = 0.53, *p* < 0.05).

### 3.6. Changes in the Serum Immune Indicators of Goat Kids

**Figure 5:**
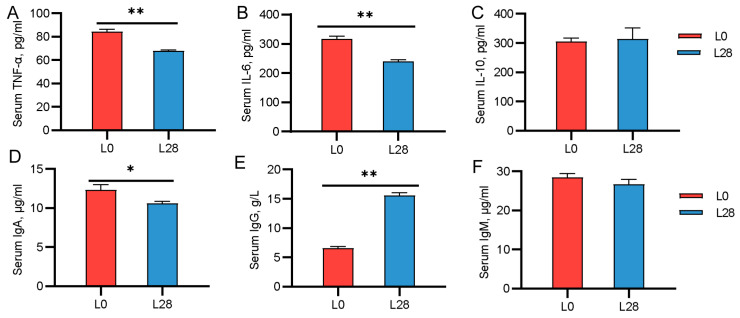
Changes in the serum immune indicators of goat kids. (**A**–**C**) Serum immune cell cytokines on day 0 and day 28 of lactation. (**D**–**F**) Serum immunoglobulin on day 0 and day 28 of lactation. L0 and L28: days 0 and 28 of lactation. *, *p* ≤ 0.05; **, *p* ≤ 0.01. *n* = 6/stage.

We also evaluated changes in serum immune factor levels in suckling kids. As shown in Figure 5, compared to L28 kids, newborn kids exhibited higher serum levels of TNF-α, IL-6, and IgA and lower levels of IgG (*p* < 0.05; Figure 5A,B,D,E). No significant differences were observed in the serum levels of IL-10 and IgM between L0 and L28 kids (*p* < 0.05; Figure 5C,F).

### 3.7. The Maternal Physiological Condition Affects the Growth and Health of the Offspring

**Figure 6:**
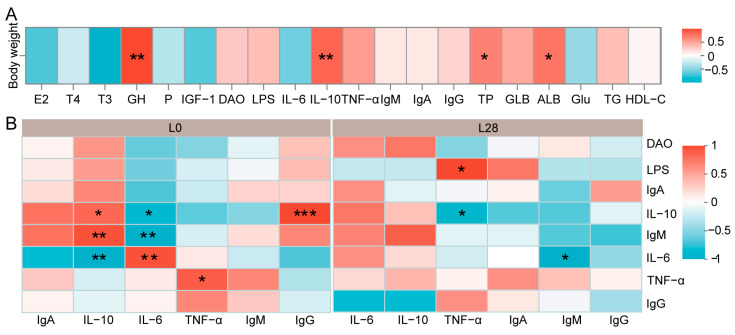
The maternal physiological condition affects the growth and health of the offspring. (**A**) Correlation between maternal physiological indicators and offspring weight. (**B**) Correlation between immune factors of kids and does. L0 and L28: days 0 and 28 of lactation. * *p* < 0.05, ** *p* < 0.01, and *** *p* < 0.001. *n* = 12/stage (six does and six kids).

We conducted a correlation analysis to assess the influence of doe serum physiological indicators on kid growth and immune function. As illustrated in Figure 6A, there was a significant positive correlation between kid body weight (Table S4) and doe serum levels of GH (r = 0.87, *p* < 0.01), IL-10 (r = 0.73, *p* < 0.01), TP (r = 0.59, *p* < 0.05), and ALB (r = 0.66, *p* < 0.05). On day 0 of lactation (Figure 6B), kid serum IL-10 was positively correlated with doe serum IL-10 (r = 0.83, *p* < 0.05) and IgM (r = 0.94, *p* < 0.01) and negatively correlated with doe serum IL-6 (r = −0.94, *p* < 0.01). Conversely, kid serum IL-6 was negatively correlated with doe serum IL-10 (r = −0.83, *p* < 0.05) and IgM (r = −0.94, *p* < 0.01) and positively correlated with doe serum IL-6 (r = 0.94, *p* < 0.01). Kid serum TNF-α was positively correlated with doe serum TNF-α levels (r = 0.89, *p* < 0.05), while kid IgG was positively correlated with doe IL-10 (r = 0.99, *p* < 0.001). On day 28 of lactation (Figure 6B), kid serum TNF-α was negatively correlated with doe serum IL-10 (r = −0.83, *p* < 0.05) and positively correlated with doe LPS levels (r = 0.83, *p* < 0.05). Kid serum IgM was negatively correlated with doe IL-6 (r = −0.89, *p* < 0.05).

### 3.8. The Microbial Composition of Multiple Body Sites of Does and Goat Kids

**Figure 7:**
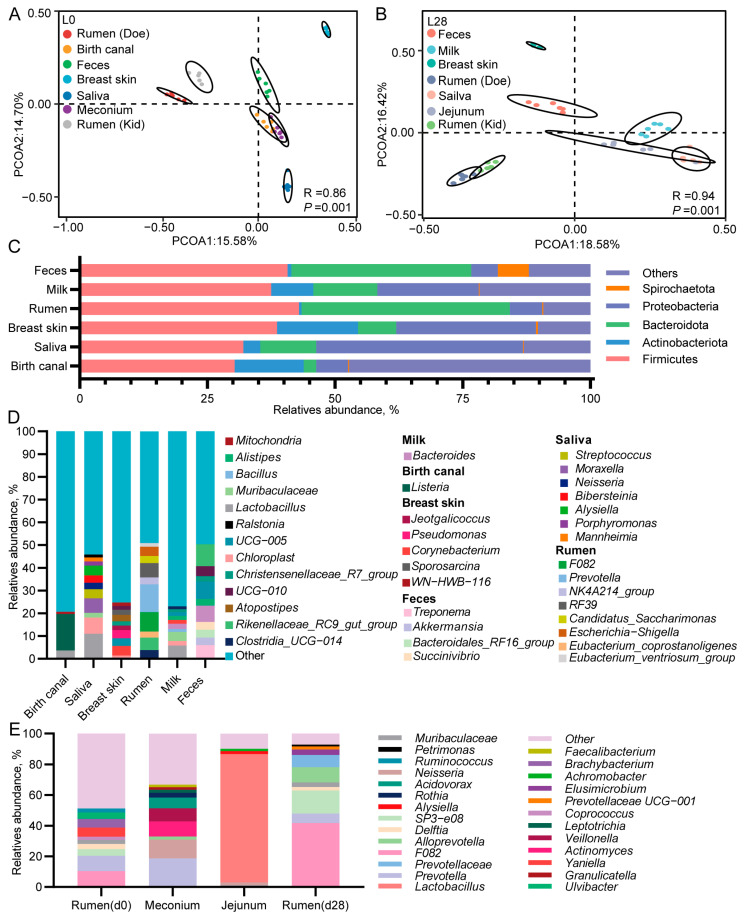
The microbial composition of multiple body sites of does and goat kids. (**A**,**B**) PCoA plot of different body sites samples. The black circle indicates the 95% confidence interval. (**C**,**D**) Relative abundance of the microbial composition at the phylum level (**C**) and genus level (**D**) across different body sites of does. (**E**), Relative abundance of the microbial composition at the genus level in the gastrointestinal of kids. *n* = 6/stage.

To investigate the sources of gastrointestinal microbiota in newborn and suckling kids, we first analyzed the microbial composition of both lactating does and kids. On both d 0 and d 28 of lactation, The PCoA and ANOSIM results revealed a largely distinct separation in the microbial composition across various body sites of both does and kids, including the birth canal, saliva, breast skin, rumen, milk, and fecal samples of does as well as the rumen and intestinal microbiomes of kids (R = 0.86, *p* =0.001; R = 0.94, *p* =0.001; Figure 7A,B). On day 0 of lactation, the microbiota from the birth canal and meconium were observed to cluster together (Figure 7A). On day 28 of lactation, the microbiota from breast milk, saliva, and the kid’s jejunum clustered together, while the rumen microbiota of both does and kids formed a cluster (Figure 7B).

The microbial composition analysis revealed that the dominant phyla in the rumen and feces of does were Firmicutes, Bacteroidetes, and Proteobacteria (Figure 7C). The dominant phyla in the birth canal, saliva, breast skin, and breast milk of does were Firmicutes, Bacteroidetes, Actinobacteria, and Proteobacteria (Figure 7C). At the genus level (Figure 7D), *Listeria* (16.08%) and *Lactobacillus* (3.6%) were the most abundant in the birth canal of does, while *Lactobacillus* (10.95%) and *Chloroplast* (7.16%) were most abundant in saliva samples. In the breast skin samples, *Pseudomonas* (3.64%) and *Corynebacterium* (4.24%) exhibited the highest relative abundance. The rumen samples primarily comprised *F082* (8.51%), *Prevotella* (12.24%), and *RF39* (6.24%). The dominant microbe in the breast milk sample was *Lactobacillus* (5.75%). In the fecal samples, the most abundant genera were *Rikenellaceae_RC9_gut_group* (9.66%), *Bacteroides* (7.14%), and *UCG-005* (7.62%).

We then analyzed the gastrointestinal microbial composition of newborn kids and 28-day-old suckling kids. We initially removed bacteria from the kids’ gastrointestinal tract that overlapped with the environmental sample to prevent environmental contamination from influencing the test data. According to Figure 7E, *F082* (10.44%), *Prevotella* (9.92%), *Yaniella* (5.99%), and *Brachybacteria* (5.63%) were the predominant genera in the rumen mucus samples of newborn kids. In the meconium samples of newborn kids, the dominant genera included *Prevotella* (18.65%), *Neisseria* (12.32%), *Actinobacteria* (9.85%), *Veillonella* (8.52%), and *Acidovorax* (7.03%). On d 28 of lactation, *Lactobacillus* (83.78%) had the highest abundance in the jejunum of kids. The dominant genera in the rumen were *F082* (41.80%), *SP3-e08* (15.80%), *AllPrevotella* (10%), *Prevotellaceae* (7.95%), and *Prevotella* (6.14%).

### 3.9. Mother-to-Infant Microbial Transmission from Different Body Sites

**Figure 8:**
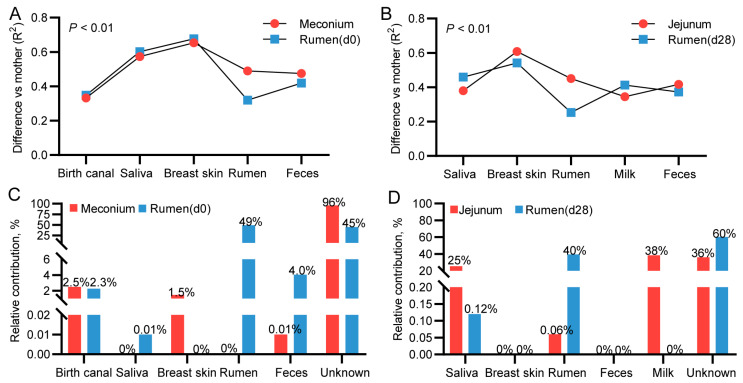
Mother-to-infant microbial transmission from different body sites. (**A**,**B**) Analysis of the association between kid and maternal microbiota composition using PERMANOVA testing. R^2^, variance explained. A smaller R^2^ value indicates a higher degree of similarity between the microbial compositions of the doe and kid. (**C**,**D**) Evaluation of the contribution of maternal microbiota to that of their offspring using Source Tracker analysis. L0 and L28: day 0 and 28 of lactation. *n* = 6/stage.

To evaluate the contribution of the maternal microbiome to the colonization of the kid gastrointestinal microbiota, we first performed a PERMANOVA test to assess the links between the microbiota in various doe body sites (including the birth canal, saliva, breast skin, milk, rumen, and feces) and the composition of the kid’s gastrointestinal microbiota. Before conducting the analysis, we excluded microbial ASVs from the samples collected from various body parts of both does and kids that overlapped with those found in the environmental sample. We found that the microbiota in the rumen mucosa of newborn kids was most similar to those in the rumen (R^2^ = 0.31) and birth canal (R^2^ = 0.35) of does (Figure 8A). The microorganisms in the meconium were most similar to those in the birth canal (R^2^ = 0.33) and feces (R^2^ = 0.47) of does (Figure 8A). On d 28 of lactation, the rumen microbiota of kids was most similar to that of does (R^2^ = 0.25) (Figure 8B). Additionally, the jejunal microbiota of 28-day-old kids was most similar to that in breast milk (R^2^ = 0.35) and doe saliva (R^2^ = 0.37) (Figure 8B).

We used Source Tracker analysis to quantify the contribution of doe microbial to the kid microbial community. As shown in Figure 8C, the rumen microorganisms of does were the primary contributors (49%) to the rumen mucosa microbiota of newborn kids. Approximately 2.5% of the microbes in the meconium originated from the birth canal, while 1.5% came from the breast skin. On d 28 of lactation, the microorganisms in the jejunum of kids were predominantly derived from breast milk (38%) and saliva (25%) (Figure 8D). Additionally, 40% of the microorganisms in the kid rumen were sourced from the doe’s rumen (Figure 8D).

### 3.10. Correlation Analysis Between Immunological Indicators and Microorganisms in Goat Kids

**Figure 9:**
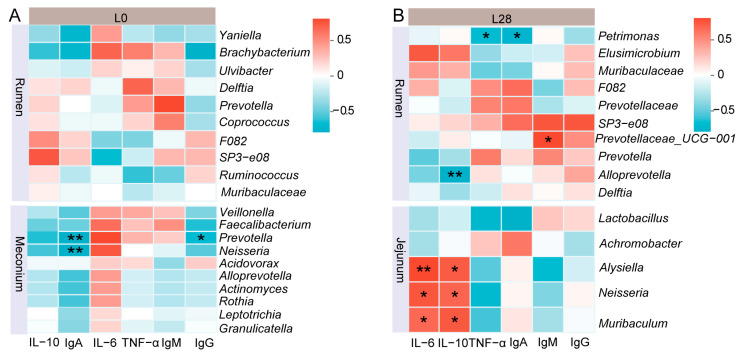
Correlation analysis between immunological indicators and microorganisms in goat kids. (**A**) Correlation analysis between rumen and meconium microbiota and serum immune factors in neonatal goats. (**B**) Correlation analysis between rumen and jejunum microbiota and serum immune factors in 28-day-old goats. L0 and L28: day 0 and 28 of lactation. * *p* < 0.05 and ** *p* < 0.01. *n* = 6/stage.

As illustrated in Figure 9, there were no significant correlation between rumen mucosal microorganisms and serum immune indicators in newborn kids (Figure 9A; *p* > 0.05). In the meconium, *Prevotella* was negatively correlated with serum IgA (r = −0.94, *p* < 0.01) and IgG (r = −0.82, *p* < 0.05), while *Neisseria* was negatively correlated with IgA (r = −0.94, *p* < 0.01). On d 28 of lactation (Figure 9B), *Petrimonas* in the kid rumen was negatively correlated with TNF-α (r = −0.83, *p* < 0.05) and IgA (r = −0.89, *p* < 0.05). Rumen *Prevotella_UCG-001* was positively correlated with IgM (r = 0.83, *p* < 0.05), and *AllPrevotella* was negatively correlated with IL-10 (r = −0.94, *p* < 0.01). Furthermore, *Alysiella*, *Neisseria*, and *Muribaculaceae* in the kid jejunum were positively correlated with IL-6 (r = 0.94, r = 0.90 and r = 0.83; *p* < 0.05) and IL-10 (r = 0.88, r = 0.84 and r = 0.89; *p* < 0.05).

## 4. Discussion

During gestation and lactation, dams must cope with a variety of challenges, including changes in tissues and organs, increased nutritional requirements, and an elevated risk of disease [37]. To address these challenges, it is essential for the hormone levels, immune function, and metabolic status of the dam to continuously adapt and change in order to maintain physiological balance and overall health. This adaptation is crucial for providing a favorable growth environment and adequate nutritional support for the offspring. Therefore, we first investigated the physiological changes in does during the mid-gestation and lactation by measuring serum hormone levels, metabolic parameters, and immune response indicators. Previous studies have yielded reliable conclusions regarding the variation of reproductive hormones in maternal throughout the reproductive cycle [38]. This study found that E2 and P levels in the serum of does increased significantly during gestation and then decreased rapidly after parturition. Zhang et al. also demonstrated that the serum levels of E2 and P in pregnant goats were significantly higher than those in lactating goats [24]. E2 and P not only stimulate the growth of uterine muscle by promoting the hyperplasia and hypertrophy of myometrial cells but also enhance blood flow to the uterus and placenta by upregulating the formation of blood vessels in both tissues [39,40,41]. The regular fluctuations in reproductive hormones observed throughout the trial indicated that the doe was in optimal pregnancy condition. Meanwhile, our research findings indicated that the serum levels of GH and T3 in does increased progressively from mid-gestation to lactation. Studies on sheep have shown that thyroid hormone levels gradually increase from mid-pregnancy, peak in late pregnancy, and remain elevated thereafter. Additionally, growth hormone concentrations rise sharply by day 120 of pregnancy, a finding that is consistent with the results of the present study [3,4]. GH not only contributes to the growth and development of the placenta by mediating cell proliferation, but it also plays a critical role in stimulating maternal gluconeogenesis and lipolysis [38,42]. From mid-gestation onward, the fetus enters a phase of rapid growth, while postdelivery, the doe must transition to a physiological state with increased energy demands to support lactation. During both of these periods, the doe’s metabolism shifts toward an active catabolic state to meet these heightened energy requirements. In this context, the sustained elevation of GH levels is likely crucial for facilitating these metabolic adaptations, thereby ensuring that the doe can effectively support both fetal development and subsequent lactation. Thyroid hormones are vital for the development of fetal tissues and organs as well as for the regulation of lung gas exchange and body temperature after birth [43,44]. Therefore, the sustained increase in serum T3 levels observed in the does in this study has significant implications for both the fetal development and the postnatal survival. We also observed that the serum levels of TP, ALB, GLB, and Glu were lower in pregnant does compared to lactating does. Furthermore, as pregnancy advanced, the levels of these four metabolites showed a consistent downward trend. The decrease in maternal serum metabolite levels during pregnancy may be attributed to several factors. First, the maternal demand for metabolites increases significantly to support fetal growth and development. Second, the expansion of blood volume during pregnancy further contributes to the relative decrease in serum metabolite concentrations [45]. As pregnancy progresses, metabolite levels gradually decline, returning to pre-pregnancy levels after delivery.

During gestation, the maternal organism undergoes a variety of physiological and metabolic changes that primarily support fetal development. However, some of these changes can also negatively impact maternal health. Specifically, during pregnancy, maternal TG levels are specifically elevated, insulin sensitivity is reduced, and inflammatory responses are elevated. These physiological changes are closely associated with increased risk of metabolic disease in the mother [46]. TGs are an essential source of nutrition for fetal growth and development, and their levels physiologically increase during pregnancy. However, abnormally high TG levels during the second and third trimesters may be linked with an elevated risk of metabolic dysfunction in mothers [47,48]. Our study found that, on d 140 of gestation, serum TG levels in pregnant does were significantly higher than at other time points. This suggests that pregnant does in late gestation may be at a higher risk of developing metabolic syndrome. To verify the idea, we proceeded to analyze changes in serum inflammatory and immune factors in does. We found that serum LPS levels in does were relatively high from mid-gestation to the first day postpartum. Additionally, from 75 to 140 days of gestation, serum levels of the anti-inflammatory factor IL-10 showed a general decreasing trend, while serum levels of the pro-inflammatory factor TNF-α exhibited a general increase. Furthermore, serum IgG levels in does were significantly reduced on day 140 of gestation. LPS, a potent inflammatory inducer, activates immune cells and promotes the release of pro-inflammatory cytokines [49]. TNF-α, a key pro-inflammatory cytokine, directly mediates inflammatory responses [50]. In contrast, IL-10 exerts anti-inflammatory effects by inhibiting the production of inflammatory cytokines, thereby promoting the resolution of inflammation [51]. Furthermore, IgG facilitates pathogen clearance by specifically binding to antigens, which indirectly alleviates the inflammatory response [52]. These findings suggest that the inflammatory response observed in late pregnancy is likely linked to elevated LPS levels, reduced IL-10 and IgG levels, and increased TNF-α levels.

During pregnancy and lactation, specific alterations in the maternal gastrointestinal microbiota and its metabolites are linked to pregnancy outcomes and lactation performance in animals [53]. Therefore, we then examined changes in the maternal rumen microbiota during pregnancy and lactation and explored how rumen microorganisms and their metabolites contribute to physiological changes in the host. This study revealed substantial differences in the rumen microbial compositions in does during pregnancy and lactation. Specifically, the abundance of Firmicutes in the rumen gradually increased from mid-gestation to day 28 of lactation. In contrast, the abundance of Bacteroidetes correspondingly decreased during this period. Members of the Firmicutes phylum are known to exhibit greater efficiency in promoting energy uptake from the host’s diet compared to Bacteroidetes. In fact, a higher Firmicutes-to-Bacteroidetes ratio has been positively correlated with increased energy uptake capacity in the host [54]. Given the increasing maternal energy demands from mid-pregnancy to lactation, the observed alterations in the proportion of rumen Firmicutes and Bacteroidetes in does are likely closely associated with the regulation of maternal energy metabolism during this period. Additionally, we found that several hormones (T3, GH, E2, P, and IGF-1), metabolic markers (Glu and TG), and immune-related indicators (TNF-α, LPS, IL-10, and IgG) in doe serum had significant associations with key rumen microbes. Notably, *Clostridia_UCG-014*, *RF39*, *Lachnospiraceae_AC2044_group*, *Eubacterium_ventriosum_group*, *Absconditabacteriales__SR1*, and *Oscillospira* exhibited the highest number of interactions with physiological indicators in doe serum, suggesting their potential roles in modulating host metabolism and immune responses. We found a gradual increase in the abundance of *Clostridia_UCG-014* from day 75 of gestation to the first day postpartum. This temporal pattern suggests that *Clostridia_UCG-014* may play a role in adapting maternal metabolism and immune function during the transition from gestation to lactation. Additionally, our study identified a significant association between *Clostridia_UCG-014* and several key factors, including T3, GH, E2, Glu, and TG in the serum of does. Although current research on the specific role of *Clostridia_UCG-014* in host hormone regulation and metabolic processes is limited, preliminary findings by Jiang et al. have suggested a potential link with the host’s lipid metabolism [55]. Based on our experimental results, we speculate that *Clostridia_UCG-014* may influence hormonal regulation and energy metabolism in does. Notably, *Clostridia_UCG-014* has been identified as a pro-inflammatory bacterium that induces host inflammation by promoting the production of lipid pro-inflammatory metabolites, such as 12-oxo-20-trihydroxy-leukotriene B4 and LysoPC [56]. Our study found a significant positive correlation between *Clostridia_UCG-014* abundance and serum TNF-α levels and a significant negative correlation with IgG levels. These findings suggest that *Clostridia_UCG-014* may contribute to maternal inflammatory responses during pregnancy and lactation, potentially through its metabolic activities. Our findings found a significant inverse association between *Oscillospira* and TNF-α levels. Previous studies have shown that *Oscillospira* may inhibit the proliferation of harmful bacteria through synergistic interactions with other beneficial bacteria, such as *Bacillus subtilis* and *Bacillus amyloliquefaciens*, thereby maintaining gut microbiota balance and reducing the occurrence of inflammatory responses [57]. This suggests that *Oscillospira* may play a protective role in modulating maternal immune responses during critical physiological periods. In this study, the *Lachnospiraceae_AC2044_group* and *Absconditabacterales_SR1* were observed to be significantly more abundant in the rumen of does on the first day of lactation. Jiao et al. reported that *Lachnospiraceae_AC2044* is negatively correlated with inflammatory markers and immunoglobulin levels in host serum [58]. Zhong et al. suggested that *Absconditabacterales_SR1* is linked to inflammatory responses in animals [59]. Our result suggests that both *Lachnospiraceae_AC2044_group* and *Absconditabacterales_SR1* were negatively correlated with the inflammatory marker LPS. These findings indicate that these microbial taxa may contribute to the suppression of inflammatory responses, potentially through the production of anti-inflammatory metabolites or interactions with host immune pathways. Despite significant correlations between *Lachnospiraceae_AC2044_group* and *Absconditabacterales_SR1* with immune factor levels observed in both the present study and previous research, the anti-inflammatory mechanisms of these taxa remain unclear. Further studies are needed to elucidate their anti-inflammatory effects. In conclusion, our study provides new insights into the role of specific rumen microbiota in modulating maternal inflammatory responses during pregnancy and lactation. It highlights the immune regulatory potential of these taxa, although the mechanisms remain unclear. These findings lay the foundation for future research to clarify the immune regulation mechanisms of these rumen microbiota and explore their therapeutic potential in improving maternal health outcomes. From mid-gestation to day 28 of lactation, the abundance of *RF39* and *Eubacterium_ventriosum_group* showed an increasing trend in does. *RF39* is primarily associated with acetate production, while *Eubacterium_ventriosum_group* is mainly associated with propionate and isobutyrate production [55,60,61]. It is important to note that than 70% of the energy requirements of ruminants are provided by VFAs, which are essential for maintaining the various physiological metabolic activities of the animal body. In this study, we also found that *RF39* and *Eubacterium_ventriosum_group* were significantly correlated with various hormones and immune factors in the serum of does, including T3, GH, TNF-α, E2, P, and LPS. Acetate serves as a precursor for cholesterol synthesis, which is essential for steroid hormone production, including E2 and P [62]. Similarly, propionate is known to influence gluconeogenesis and insulin sensitivity, thereby indirectly modulating metabolic processes and hormone secretion [63]. Furthermore, VFAs can act as signaling molecules, interacting with G-protein-coupled receptors on host cells to regulate immune responses and inflammatory pathways, such as those involving TNF-α [64]. These findings suggest that *RF39* and *Eubacterium_ventriosum_group* may contribute to the regulation of maternal physiological metabolism through their metabolites, particularly VFAs. Based on the above results, we speculate that microbial metabolites, VFAs, play a crucial role in the microbial regulation of host physiological activities. To investigate this further, we analyzed the changes in VFAs content in the rumen of does.

Acetate is the key energy substrate for ruminant metabolism [65]. In our study, we observed an overall increase in rumen acetate levels from mid-pregnancy to lactation, which aligns with the heightened energy demands during these critical stages. Acetate is primarily utilized for lipogenesis and muscle growth, both of which are essential for the development of maternal adipose tissue and muscle mass during pregnancy, and for milk production during lactation [65]. The increased acetate production in the rumen can therefore be viewed as a compensatory mechanism that ensures an adequate energy supply to both the doe and the developing fetus. Additionally, we identified a positive correlation between rumen acetate content and serum GH and T3 concentrations. Both GH and T3 play crucial roles in the development of the placenta, fetal tissues, and the overall metabolic regulation during pregnancy and lactation [38,42,43,44]. The increase in acetate production in the rumen appears to support these hormonal changes, contributing to the metabolic adaptations required for fetal growth and maternal tissue development.

Propionate plays a critical role in energy metabolism, particularly as a key precursor for gluconeogenesis in the liver, where it is converted into Glu [65]. Glu is the major energy source for the fetus. Our study revealed a significant positive relationship between rumen propionate levels and serum Glu concentrations, further emphasizing the critical role of propionate in meeting the energy demands of the fetus.

The transition from pregnancy to lactation brings shifts in VFA production and utilization. During pregnancy, the increased demand for energy substrates like acetate and propionate supports fetal growth. Upon entering lactation, the demand for energy shifts to milk production, which requires an increased supply of both acetate for fat synthesis and propionate for glucose production. The dynamic changes in VFA production during pregnancy and lactation reflect the adaptation of ruminant metabolism to meet the increased energetic demands of both the doe and the developing fetus, promoting optimal growth and development.

Moreover, our findings provide new insight into the role of rumen microorganisms in regulating maternal physiological functions and metabolism. Future research should focus on elucidating the specific biochemical pathways through which VFAs influence hormone and immune responses. Additionally, further investigation is needed to explore how the maternal microbiome can be modulated to enhance health outcomes in ruminants. The health and microbial community composition of the dam are crucial for the growth, immune system maturation, and gastrointestinal microbiota colonization of her offspring. In this study, we observed that serum GH, immune regulatory factor IL-10, and protein metabolism-related indicators TP and ALB in lactating does were positively correlated with kid weight. This finding aligns with Tygesen et al., who indicated that prenatal nutritional restriction in ewes leads to a decrease in plasma IGF-1 concentration, impaired lactation function, and, consequently, relatively lower body weight in offspring [66]. Similarly, the study by Ramos Lobo et al. also confirmed that the physiological changes commonly observed during pregnancy and lactation are essential for the normal development of offspring [67]. Furthermore, our research identified a significant association between immune factors in lactating does and their kids, particularly immune factors such as IL-10, IL-6, TNF-α, and IgG in the serum of newborn kids, which were significantly correlated with at least one immune factor in the doe’s serum. This finding aligns with Liu et al., who reported a positive correlation between IL-10 levels in piglet and sow serum as well as a positive correlation between TNF-α in piglet serum and IL-6 and TNF-α in sow serum [1]. The dam transmits immune factors to her offspring through both blood and milk, providing essential protection for their early health [29,68]. This mechanism may account for the observed correlation between maternal and fetal serum immune markers in this study.

Further research has shown that the maternal microbiota plays a pivotal role in the development of the offspring’s immune system. Offspring acquire beneficial bacteria, such as *Bifidobacterium* and *Lactobacillus*, from the maternal milk and amniotic fluid through intrauterine swallowing and breastfeeding [69]. These maternally derived microbes and their metabolites are vital for the development and regulation of both maternal and offspring immune systems. For instance, *Bifidobacterium* produces acetate and lactic acids, while *Lactobacillus* generates VFAs and bacteriocins, which collectively contribute to immune modulation [69,70,71]. Importantly, changes in the maternal microbiota can significantly alter the maternal microbial environment and metabolic state, thereby influencing the offspring’s microbial colonization and immune status [72,73]. Wang et al. demonstrated that fecal microbiota transplantation (FMT) in dams, following LPS administration to both dams and their offspring, markedly increased the abundance of probiotics (such as *Lactobacillus* and *Clostridium*) in the dams’ guts, with a corresponding rise observed in the offspring’s gut microbiota. FMT also effectively mitigated LPS-induced intestinal damage and reduced serum levels of pro-inflammatory cytokines in both dams and offspring. This protective effect is likely due to metabolites (e.g., organic acids, enzymes, and antimicrobial peptides) produced by the fecal microbiota [74]. These findings highlight that the maternal microbiota, through vertical transmission and metabolite interactions, plays a crucial role in the synchronized regulation of maternal and offspring immune systems. This microbiome–metabolism–immunity cascade mechanism may explain the correlation observed between maternal and offspring serum immune markers in this study. In early life, the immune system of animals undergoes rapid development and is profoundly influenced by the microbiome [75]. However, our understanding of initial microbial colonization and community development in young animals remains limited. Against this backdrop, we examined the role of maternal microbiota in the colonization of gastrointestinal microbiota in newborn and suckling kids. Additionally, we also explored how early gastrointestinal microbiota colonization in kids influence the development of immune system. In this study, the birth canal microbiome contributed the most to the meconium microbiome composition. The primary source of microbiota in the rumen of newborn kids is the doe’s rumen microbiota. At same time, our study found that newborn kids possess a distinct microbial system that is notably different from the maternal microbiome. After minimizing potential environmental interference, we observed that 96% of the microorganisms in the meconium and 45% of those in the rumen mucus of newborn kids were not directly related to the microbial communities found in various parts of the maternal body. This indicates that these distinct microbial communities may be related to the uterine environment where the fetal kids develop. However, although previous studies have examined the possibility of microbial colonization in the uterus, there is ongoing debate about whether the uterine environment is the true source of these microbes [76]. This controversy arises from the limitations of current detection technologies and challenges in controlling environmental contamination. Therefore, further research is needed to provide a conclusive answer.

We found that the primary source of microbiota in the rumen of 28-day-old kids is the maternal rumen microbiota. Jin et al. also revealed that 31.5% of the microorganisms found in the rumen of lambs were closely related to those present in the rumen of ewes [77]. While the mechanism of maternal transmission of rumen microbiota to offspring is still under investigation, previous studies suggest that this transfer may occur through behaviors such as licking [78]. Specifically, a study on dairy cows showed that the microbial composition in the saliva of dairy cows were highly similar to those in their rumen [79]. Zhuang et al. further observed that cows habitually licked their newborn calves after delivery, and they speculated that this licking behavior could facilitate the transfer of rumen microorganisms to the calf via saliva-mediated pathways [80]. Therefore, it is plausible that similar licking behavior could serve as a potential pathway for the transmission of rumen microorganisms from the doe to the kid, although further experimental verification is needed to establish this direct link. However, the exact pathway through which newborn kids acquire their rumen microbiome from the doe’s rumen is not yet fully understood and needs further investigation. Mothers can transfer microorganisms to their offspring through suckling and licking behaviors [78]. The results of this study support this notion, indicating that the primary sources of the jejunum microbiota in 28-day-old kids were breast milk (38%) and doe saliva (25%). Furthermore, the dominant bacteria *F082* and *Prevotella* in the rumen of kids, along with *Lactobacillus*, the dominant bacterium in the jejunum of 28-day-old suckling kids, are also prevalent in the rumen or milk of does. These results emphasize the importance of the maternal microbiota in shaping the kid’s gastrointestinal microbiota development. Notably, the colonization of *Prevotella* and *Lactobacillus* in the gastrointestinal tract of young ruminants significantly improves microbial composition and fermentation efficiency, thereby promoting host growth and development. [81,82]. Mantrana et al. found that the maternal diet during pregnancy influences the initial microbial colonization of newborns by modulating the maternal microbiota. This mechanism leads to significant differences in the risk of overweight at 18 months of age among children born to mothers with different dietary patterns during pregnancy. Additionally, their study highlighted that the composition of the maternal microbiota serves as an important biomarker for predicting the risk of overweight in offspring at 18 months [83]. These findings improve our understanding of maternal microbial transmission and its long-term health effects on offspring.

The survival of young animals is closely linked to the establishment of their early immune defense mechanisms, which are significantly influenced by their microbial composition [84,85]. To explore the relevance between microbial composition and immune responses, we determined correlation between dominant bacterial genera in the gastrointestinal tract of kids and their serum immune factors. It was found that the dominant genera *Prevotella* and *Neisseria* in the meconium were significantly negatively correlated with serum IgA levels. Additionally, in 28-day-old kids, the dominant genera *Petrimonas*, *Prevotella_UCG-001*, and *AllPrevotella* in the rumen showed notable correlations with serum immune factors. Furthermore, *Alysiella*, *Neisseria*, and *Muribaculaceae* in the jejunum of kids exhibited a positive correlation with the levels of Interleukin. Previous studies have reported associations between *Prevotella*, *Neisseria*, *Prevotella_UCG-001*, *AllPrevotella*, and *Muribaculaceae* and host immunomodulation [86,87,88]. Wang et al. also found a significant relationship between serum immune factors and the gut microbiota in suckling piglets [74]. These findings collectively emphasize the important role that microorganisms colonizing the gastrointestinal tract of young animals play in the development of the host immune system. Our study revealed a key finding: compared to the number of significant correlations between serum immune indicators in does and lactating kids on day 28 of lactation, there was a higher number of significant correlations between serum immune factors in does and newborn kids on day 0 of lactation. Furthermore, there was no significant correlation between the rumen microbiota and serum immune indicators in newborn kids, and only a few meconium microbiotas were significantly correlated with serum immune factors. However, on day 28 of lactation, the quantity of significant associations between the gastrointestinal microbiota and serum immune indicators in kids increased substantially. Based on these observations, we speculate that in newborn kids, serum immune factors primarily originate from vertical transmission from the mother. In contrast, for 28-day-old suckling kids, the colonization of the gastrointestinal microbiota appears to be a critical factor in modulating the immune development and maturation of the host.

## 5. Conclusions

Our study indicates that from mid-gestation to lactation, does undergo significant changes in physiological metabolism, immune status, and rumen microbiota. We identified potential associations between these physiological changes and the rumen microbiome, thereby providing a scientific basis for a deeper understanding of does’ health and production performance. This research contributes to a deeper understanding of the complex relationship between the doe’s physiological processes and microbiome composition, shedding light on the mechanisms through which these factors influence the growth and immune function of both newborn and lactating kids. Our study further emphasizes the essential role of the microbiome in various regions of the doe’s body in facilitating the early colonization of the kid’s gastrointestinal microbiome, a process that is integral to the subsequent development of the kid’s immune system. These findings advance our understanding of the maternal microbiome’s influence at specific physiological stages and offer new insights into the mechanisms of microbiome transmission between does and kids as well as its impact on kid immune function.

Furthermore, the identification of key microbiome dynamics could inform strategies to enhance offspring growth and immune function, such as adjusting maternal diets or microbiome-modifying interventions during key stages of pregnancy and lactation. These strategies may help optimize the health of both does and their kids, improving overall productivity in farming operations. Future studies should explore targeted interventions in the maternal microbiome, such as probiotics or diet modifications, and their potential to further enhance offspring health outcomes.

In conclusion, this study not only advances our understanding of the maternal microbiome’s role at critical physiological stages but also lays the groundwork for future research aimed at optimizing farm management practices, improving animal health, and enhancing the overall productivity of livestock systems.

## Figures and Tables

**Table 1:** Changes in the level of volatile fatty acids in the rumen of doe during mid-gestation and lactation.

Items	G75	G105	G140	L0	L28	*p*-Value
Acetate (mmol/L)	55.64 ± 3.84 ^c^	74.91 ± 1.41 ^ab^	62.4 ± 10.12 ^bc^	74.01 ± 4.79 ^ab^	78.83 ± 8.93 ^a^	0.03
Propionate (mmol/L)	17.27 ± 0.50	18.59 ± 3.67	14.92 ± 1.20	21.41 ± 2.87	20.81 ± 3.71	0.19
Butyrate (mmol/L)	2.98 ± 0.41	3.14 ± 0.62	2.3 ± 0.19	2.76 ± 0.43	3.14 ± 0.47	0.36
Isobutyrate (mmol/L)	6.13 ± 1.45	5.58 ± 0.85	6.36 ± 0.96	5.88 ± 0.55	7.18 ± 1.09	0.61
valerate (mmol/L)	1.05 ± 0.23	0.89 ± 0.06	0.87 ± 0.03	0.88 ± 0.04	0.94 ± 0.06	0.51
Isovalerate (mmol/L)	4.21 ± 1.25	4.04 ± 0.88	4.64 ± 0.84	4.18 ± 0.53	5.19 ± 0.93	0.72
TVFA (mmol/L)	94.03 ± 7.61	107.15 ± 4.99	84.73 ± 1.96	109.13 ± 15.89	116.08 ± 22.45	0.19

G75, G105, and G140: days 75, 105, and 140 of gestation; L0 and L28: days 0 and 28 of lactation. Different letters represent significant differences (*p* < 0.05). *n* = 6/stage.

## Data Availability

Data are contained within the article and Supplementary Materials.

## Supplementary Materials

The following supporting information can be downloaded at: https://www.mdpi.com/article/10.3390/ani15070954/s1, Figure S1: Rarefaction curve analysis; Table S1: Composition and nutrient levels of experimental diets (DM basis) %; Table S2: Differences of rumen microbial structure in doe during mid-gestation and lactation; Table S3: Correlation between differential serum index and doe rumen volatile fatty acids; Table S4: Goat kid body weight at 0 and 28 days of age (kg).
