# Supplementary material for: Physiological and Microbial Community Dynamics in Does During Mid-Gestation to Lactation and Their Impact on the Growth, Immune Function, and Microbiome Transmission of Offspring Kids
Source: Animals (Basel). 2025 Mar 26;15(7):954. doi: 10.3390/ani15070954 (PMC11987885; doi:10.3390/ani15070954)
Supplement: Supplementary file 1 [file animals-15-00954-s001.zip › animals-3502290-supplementary.pdf]

animals

MDPI

Supplementary Table S1. Composition and nutrient levels of experimental diets (DM basis) %

Items	Diet
Ingredients	
Corn	20.00
Soybean meal	19.00
Corn straw	50.00
Wheat hay	1.00
concentrate supplement[a]	8.50
$NaHCO_3$	0.25
Salt	0.25
Premix[b]	1.00
Total	100.00
Nutrient Levels[c]	
ME, MJ/kg	9.21
CP	13.97
EE	3.60
Ash	5.61
NDF	43.81
ADF	25.70

[a]The doe concentrate supplement containing corn, soybean meal, cottonseed meal, Corn husk, urea Calcium hydrogen phosphate, NaCl and stone powder was purchased from Zhengda (Zhengda, Hohhot, China).
[b]Provided per kilogram of premix: vitamin A 300000 IU/kg; vitamin D3 85000 IU/kg; vitamin E 1600 IU/kg; vitamin B1 20 mg/kg; vitamin B2 55 mg/kg; vitamin B6 12 mg/kg; niacin 240 mg/kg; pantothenate 120 mg/kg; folic acid 9 mg/kg; biotin 3 mg/kg； Fe 600 mg/kg; Cu 200 mg/kg; Zn 1200 mg/kg; Ca 300 mg/kg; P 25 mg/kg.
[c]ME was calculated value, while others were all measured values.

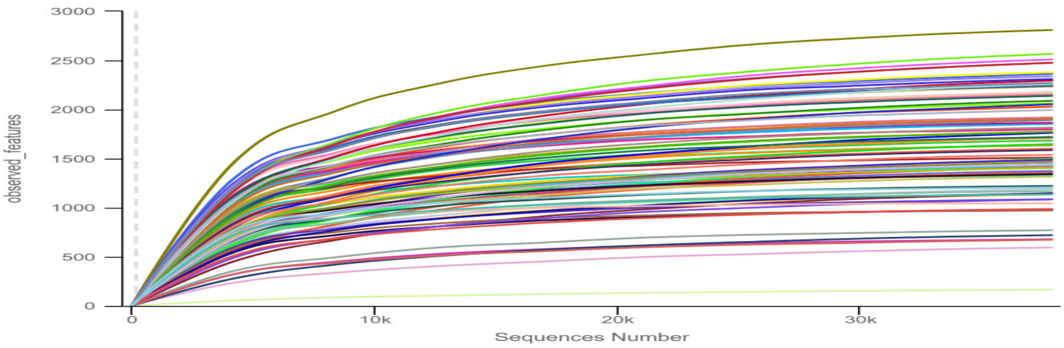

Supplementary Figure S1. Rarefaction curve analysis.

Supplementary Table S2. Differences of rumen microbial structure in doe during mid-gestation and lactation

Comparison	R (Anosim)	*P*-Value	A (MRPP)	*P*-Value
G75:G105	0.03	0.46	0.00	0.53
G75:G140	0.62	0.04	0.04	0.02
G75:L0	0.57	0.01	0.03	0.01
G75:L28	0.70	0.01	0.06	0.02
G105:G140	0.25	0.09	0.03	0.05
G105:L0	0.37	0.01	0.02	0.02
G105:L28	0.51	0.01	0.05	0.02
G140:L0	0.22	0.09	0.00	0.35
G140:L28	0.29	0.05	0.02	0.12
L0:L28	0.39	0.02	0.03	0.02

G75, G105 and G140, day 75, 105 and 140 of gestation; L0 and L28, day 0 and 28 of lactation. R-values and A-values greater than 0 indicate that the differences between groups are greater than the differences within groups.

Supplementary Table S3. Correlation between differential serum index and doe rumen volatile fatty acids

Items	Acetate	Propionate	Butyrate	Isobutyrate	Valerate	Isovalerate	TVFA
E2	-0.34	-0.59*	-0.37	-0.24	-0.37	-0.26	-0.57*
P	-0.17	-0.24	0.18	-0.08	-0.02	-0.06	-0.18
T4	0.13	-0.09	0.23	0.01	0.15	0.05	0.03
T3	0.44*	0.39	-0.08	0.16	0.06	0.12	0.27
GH	0.47*	0.44*	0.18	0.40	0.31	0.37	0.29
IGF-1	0.2	-0.04	0.08	0.52*	0.36	0.51	-0.24
DAO	-0.06	0.01	0.25	0.37	0.19	0.40	0.01
LPS	-0.29	-0.49*	-0.05	-0.17	-0.07	-0.18	-0.32
IL-6	-0.24	-0.24	0.28	0.11	0.06	0.20	-0.15
IL-10	-0.06	-0.01	0.07	0.06	0.08	0.06	0.11
TNF-α	0.35	-0.01	-0.09	0.30	0.20	0.28	0.01
IgM	-0.4	-0.18	-0.18	0.18	0.03	0.16	-0.33
IgA	-0.05	0.36	0.31	0.31	0.43	0.34	0.40
IgG	-0.24	0.04	0.19	-0.46	-0.11	-0.40	0.17
TP	0.26	0.11	0.52*	0.01	0.20	0.04	0.38
GLB	0.18	0.04	0.48	-0.03	0.22	-0.01	0.33
ALB	0.27	-0.01	0.37	0.13	0.16	0.15	0.19
Glu	0.16	0.54*	0.16	0.06	0.24	0.09	0.53*
TG	-0.34	-0.63*	-0.20	-0.05	-0.29	-0.05	-0.65**
HDL-C	0.18	0.01	0.04	-0.21	-0.11	-0.27	0.09

Supplementary Table S4. Goat kid body weight at 0 and 28 days of age (kg)

Items	0 d	28 d
body weight	3.28±0.41	8.28±1.06

L0 and L28, day 0 and 28 of lactation.
